# Laparoscopic Sleeve Gastrectomy in Situs Inversus Totalis: A Case Report

**DOI:** 10.7759/cureus.62610

**Published:** 2024-06-18

**Authors:** Hamad S Alsubaie, Wadha S AlOtaibi

**Affiliations:** 1 Department of Surgery, College of Medicine, King Saud University, Riyadh, SAU; 2 Department of General Surgery, King Saud University Medical City, Riyadh, SAU

**Keywords:** situs inversus abdominals, situs inversus totalis, situs inversus, bariatric surgery, laparoscopic sleeve gastrectomy, obesity

## Abstract

Obesity prevalence is increasing with the modern lifestyle. Bariatric surgery is an excellent method to sustain weight reduction and the most commonly performed surgery is laparoscopic sleeve gastrectomy (LSG). The laparoscopic approach can be challenged in certain conditions such as situs inversus totalis (SIT). We report a 38-year-old gentleman with class II obesity known to have SIT. After complete preoperative preparation, we performed LSG with no complications. The main difficulty of performing any surgical procedure for SIT patients is the reversed anatomy. It is essential to highlight the importance of anatomy for surgeons. Proper preoperative anatomy assessment along with the surgeon's experience is the key element to perform LSG or any bariatric laparoscopic procedure in rare conditions such as SIT.

## Introduction

Obesity prevalence is increasing worldwide with the modern lifestyle [1]. Bariatric surgery is an excellent method to sustain weight reduction when indicated and with good patient selection [2]. Laparoscopic sleeve gastrectomy (LSG) is the most performed bariatric surgery accounting for almost 50% of all the practiced bariatric surgeries [2]. LSG has a good weight reduction profile especially when combined with behavioral and nutritional counseling [3-6].

The laparoscopic approach in bariatric surgeries can be challenged in certain conditions such as situs inversus totalis (SIT) [3]. This congenital condition is a transposition of the thoraco-abdominal organs as opposed to their regular position, in which major organs are reversed [7]. The first reported laparoscopic procedure in SIT patients was a laparoscopic cholecystectomy reported in 1991 [8]. Thereafter, a variety of techniques were reported in the literature, which included but not limited to mirror placement of laparoscopic ports, referral of patients to left-handed surgeons, considering single-port surgery, and depending on thorough preoperative imaging [9].

## Case presentation

We report a 38-year-old gentleman with class II obesity and a body mass index of 36.24. He is a known case of hypothyroidism on medication and is known to have SIT. Also, he had undergone patch repair of a ventricular septal defect six years ago. He has been a smoker for three years now. The patient reported a long history of failure of achieving substantial and durable weight loss despite following up with a dietitian for dietary plans, a trial of medical therapy by an endocrinologist, and also underwent gastric balloon placement five years back.

**Figure 1:**
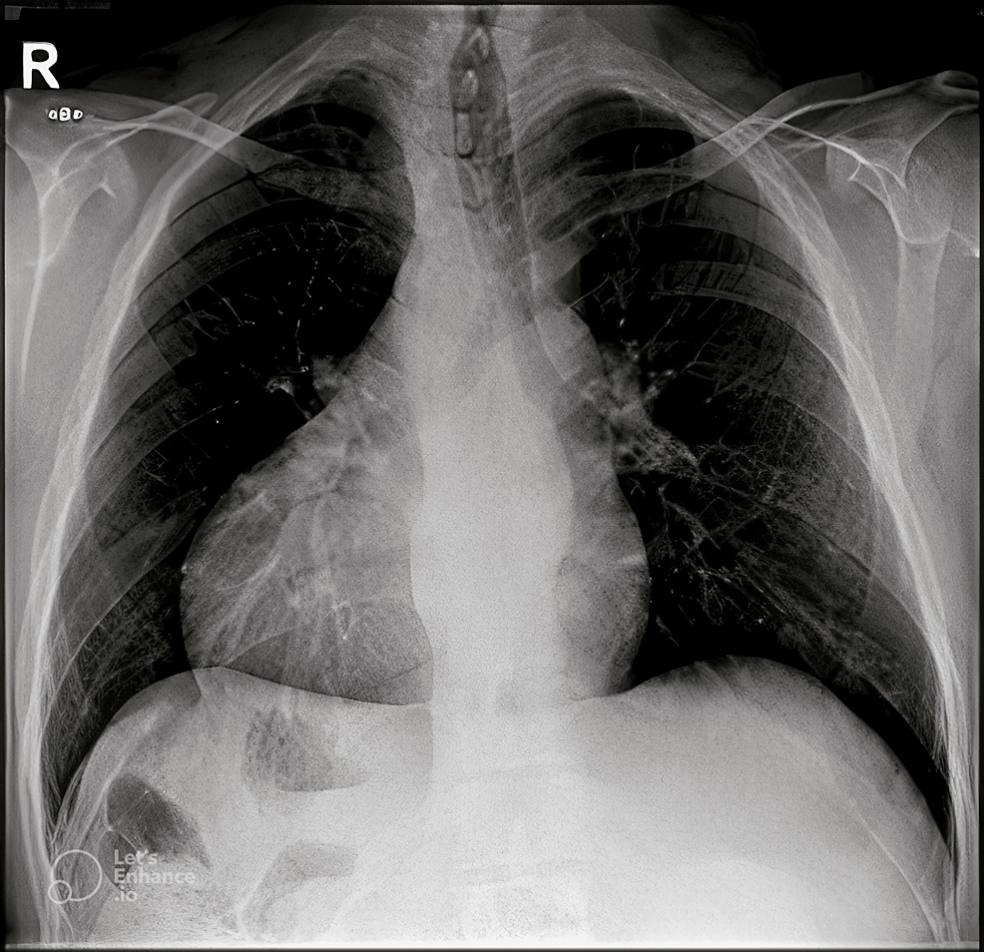
Chest X-ray showing dextrocardia with air fluids levels in the right upper quadrant of the abdomen

He was admitted electively for a thorough preoperative assessment and preparation. For cardiologic clearance, he underwent electrocardiogram (ECG) and transthoracic echocardiogram (TTE). His ECG showed sinus rhythm with first-degree atrioventricular (AV) block, right atrial enlargement, left axis deviation, and T-wave abnormality, concerning for anterior ischemia. On the other hand, his TTE revealed normal size and function of the left ventricle, no residual leak, and an ejection fraction of more than 55%. He also underwent a chest X-ray with the finding of dextrocardia (Figure 1).

**Figure 2:**
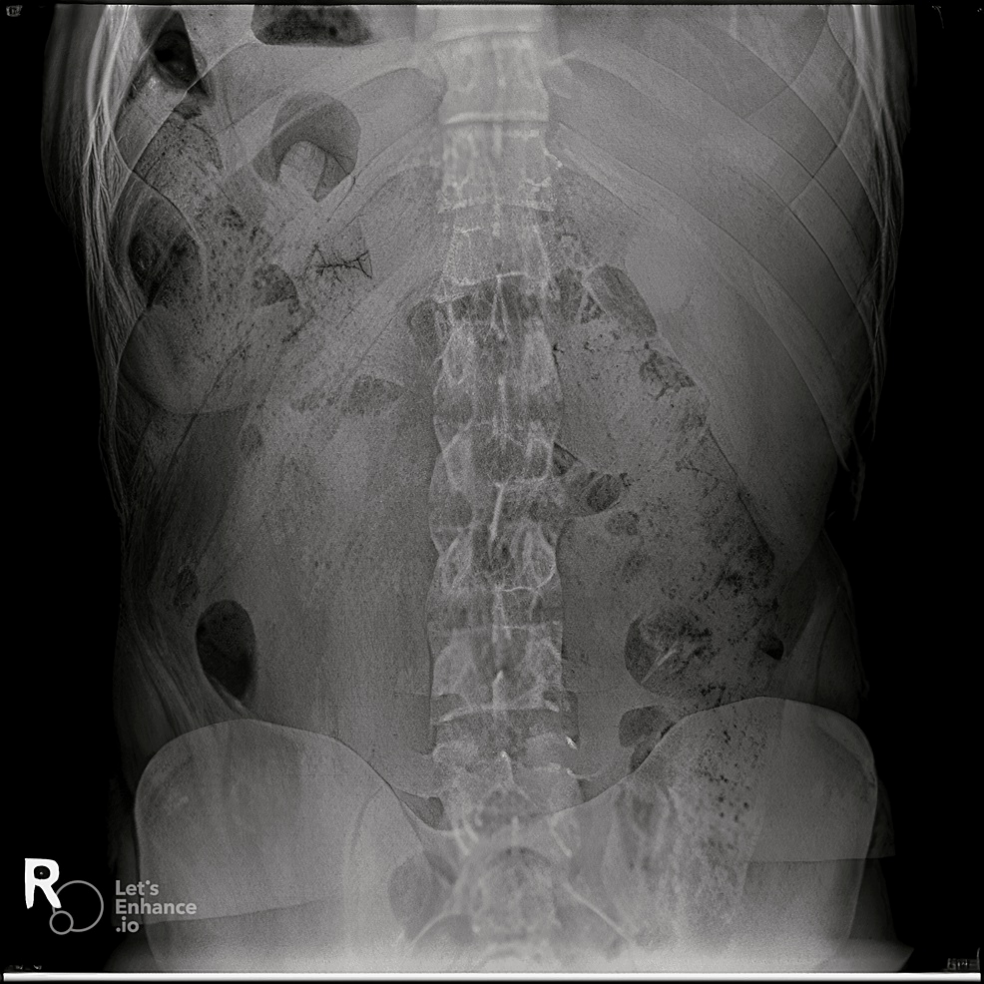
Abdominal X-ray showing mirror presentation of the intra-abdominal organs.

**Figure 3:**
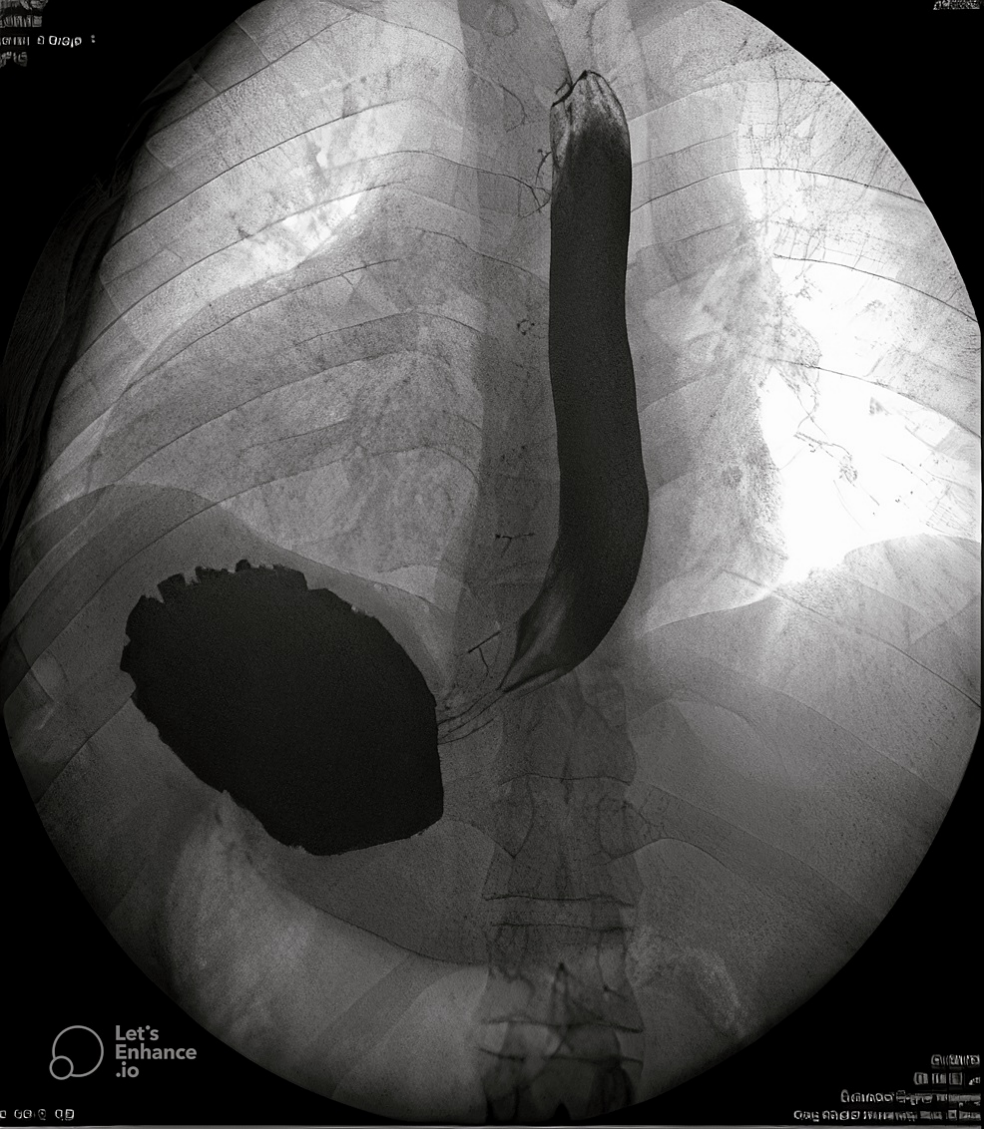
Fluoroscopy barium swallow showing mirror presentation of the intra-abdominal organs.

**Figure 4:**
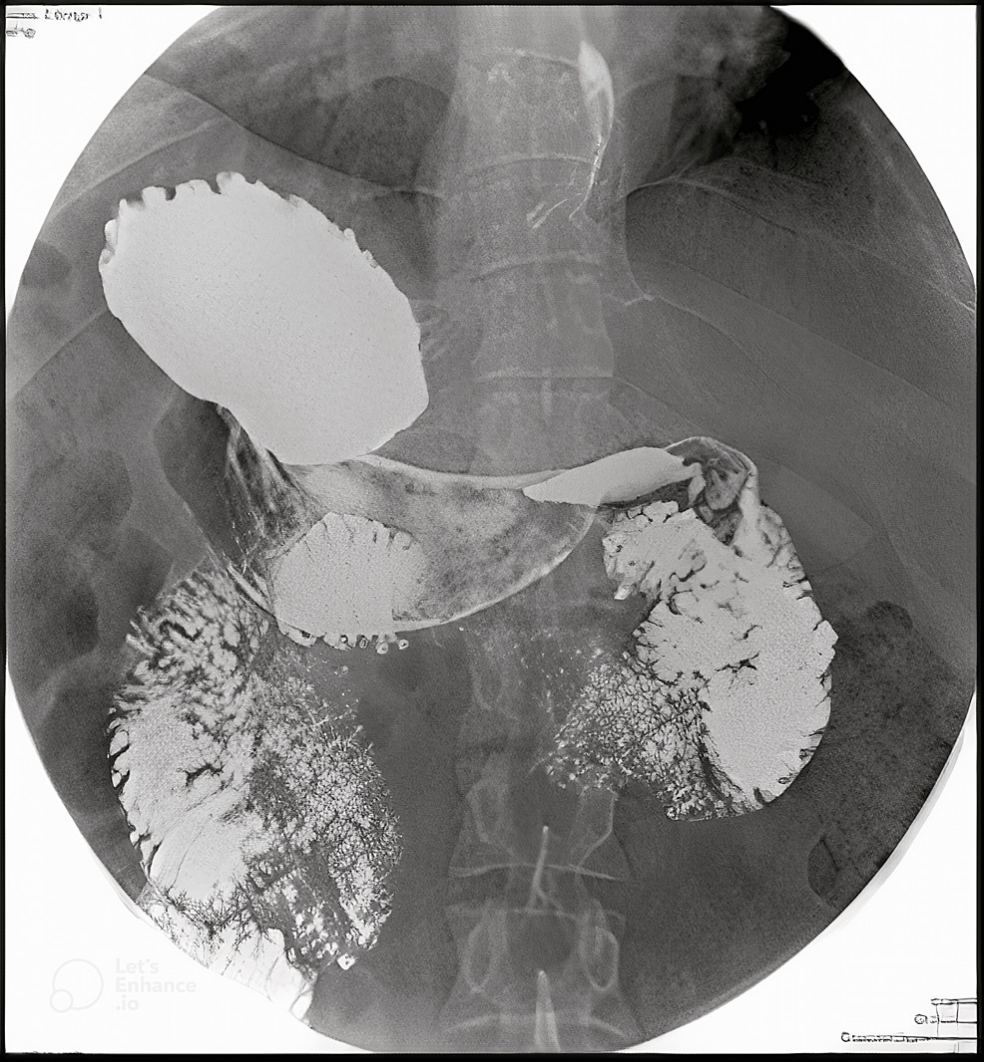
Fluoroscopy barium swallow showing mirror presentation of the intra-abdominal organs.

The patient was declared fit for surgery by the cardiologist. To complete his preoperative planning we needed to delineate his anatomy, so we decided to do an X-ray abdomen, X-ray fluoroscopy barium swallow and ultrasound (US) of the abdomen. The X-ray abdomen showed air fluids levels in the right upper quadrant and the X-ray fluoroscopy was unremarkable but for a complete situs inversus with reversed anatomical positions (Figures 2-4).

Also, the US abdomen showed no pathology and reported left-sided liver, right-sided spleen, no gallbladder stones or biliary dilatation, no hydronephrosis or obvious renal stones, and no ascites could be appreciated.

**Figure 5:**
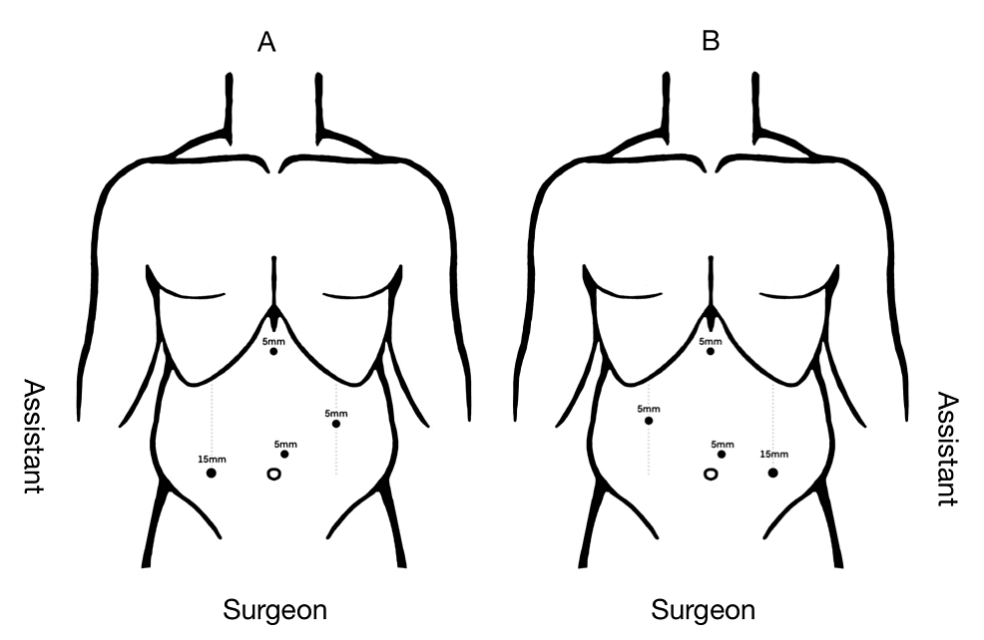
Laparoscopic sleeve gastrectomy (LSG) trocar sites (A) Patient with regular anatomy. (B) Patient with situs inversus totalis (SIT). Designed by Najd AlZahrani, medical student at King Saud University, Riyadh, Saudi Arabia.

The perioperative course was unremarkable. We performed LSG, the patient's position was split-leg position, French position (thighs in abduction with the surgeon positioned between the legs) as of all our regular cases. Although the surgeon is right-handed, he can work with both hands. We inserted four trocars, three 5mm and one 15mm trocar. We introduced pneumoperitoneum through veress needle on left upper quadrant, then 5mm optic trocar inserted supraumbilical and slightly to the left, 5mm subxiphoid trocar inserted, another 5mm trocar inserted on the right mid-clavicular line around five fingerbreadth above the umbilical level, and finally 15mm trocar inserted on the left mid-clavicular line at the level of the umbilicus (Figure 5).

**Figure 6:**
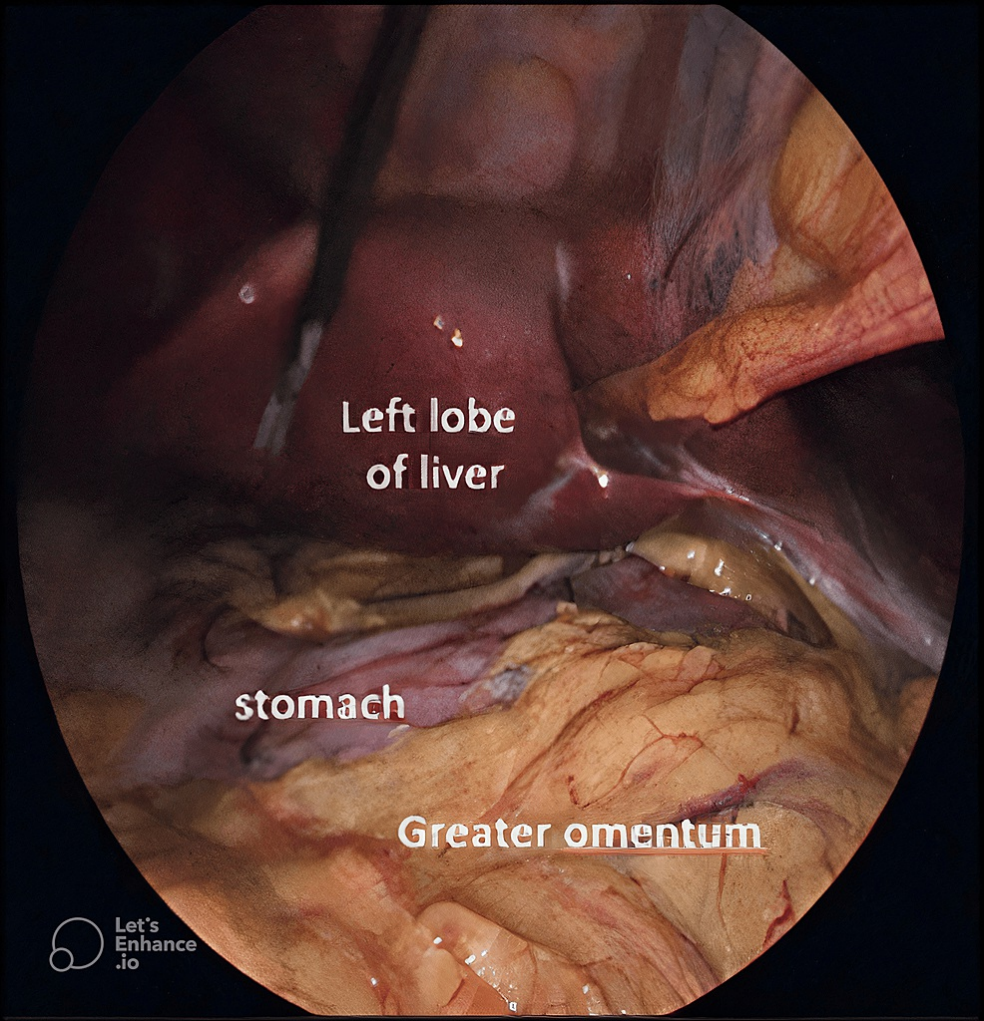
Laparoscopic view of mirror presentation of the intra-abdominal organs.

The intra-operative findings were similar to the pre-operative findings and no complications were encountered (Figure 6).

We report no prolongation of operative time, all cases performed on the same day had approximately similar operative time. The patient's postoperative course was uneventful, and he was discharged on day 1 postoperative. The patient presented to our clinic for the first follow-up, two weeks post-operative. He was doing well with no active complaint. He was following our post-operative protocol as explained to him before the discharge. Upon examination the wounds healed well and his abdomen is soft. He lost about 7 kg in the first two weeks. The pathology report revealed mild chronic active gastritis, helicobacter like organisms, and no signs of metaplasia or malignancy. The patient received clarithromycin-based therapy for 14 days post-operative. And lastly, three months after surgery, the patient presented to our clinic for a regular follow-up with a BMI of 31.

## Discussion

Situs inversus totalis (SIT) is a rare congenital condition where organ function is generally normal, and as in our patient, this condition is frequently associated with respiratory or cardiovascular anomalies [7]. The main difficulty of performing any surgical procedure for SIT patients is the reversed anatomy. It is essential to highlight the importance of anatomy for surgeons [3-6]. Laparoscopic bariatric surgery in patients with SIT was reported in 27 cases in the literature (Table 1). The predominant procedure that was performed on those patients was LSG on a total of 16 cases, six cases underwent laparoscopic Roux-en-Y gastric bypass, five cases underwent laparoscopic adjustable gastric banding, only one case had laparoscopic sleeve gastrectomy with duodenojejunal bypass, and also one case had single incision laparoscopic sleeve gastrectomy (Table 1).

**Table 1:** A brief review of the studies of bariatric surgeries with situs inversus totalis BIB: bioenteric intragastric balloon, BMI: body mass index, CT: computed tomography, DM: diabetes mellitus, ECG: electrocardiography, HT: hypertension, LAGB: laparoscopic adjustable gastric banding, LC: laparoscopic cholecystectomy, LRYGB: laparoscopic Roux-en-Y gastric bypass, LSG: laparoscopic sleeve gastrectomy, NA: not available, OSAS: obstructive sleep apnea syndrome, SILSG: single incision laparoscopic sleeve gastrectomy, USG: abdominal ultrasonography, VFSE: video fluoroscopic swallowing exam, EGD: esophagogastroduodenoscopy, DJB: Duodeno-Jejunal bypass.

Principle Author -Year (Reference)	Age/ Gender	Body Mass Index Pre-operation (kg/m^2^)	Preoperative Diagnostic Method	Operation Time (Mean Operation Time)	Previous Operation	Surgical Procedure	Postoperative Complications
Aziret M - 2016 [3]	54/F	48 kg/m^2^	X-ray chest	105 min	Open cholecystectomy	LSG	No
Salerno A - 2018 [5]	41/M	46.4 kg/m^2^	NA	45 min	LSG	LSG	No
Froylich D - 2018 [6]	47/F	51 kg/m^2^	VFSE/X-ray chest	62 min	NA	LSG	No
Stier C - 2014 [10]	39/M	44 kg/m^2^	USG/X-ray chest/gastroscopy/ECG	76 min (50–93)	No	LRYGB	No
Stier C - 2014 [10]	51/F	54.2 kg/m^2^	USG/X-ray chest/gastroscopy/ECG	61 min (16–87)	No	LSG	No
Deutsch - 2012 [11]	39/F	42 kg/m^2^	Abdominal CT	NA	Open Gastric Banding	LSG	Suture Line Leakage
Ahmed AR - 2006 [12]	47/F	58.1 kg/m^2^	ECG/X-ray chest/CT scan	160 min (105)	No	LRYGB	No
Wittgrove AC - 1998 [13]	38/F	47.8 kg/m^2^	ECG/X-ray chest	300 min (159)	No	LRYGB	No
Tsepelidis D - 2015 [14]	51/F	43 kg/m^2^	NA	120 min (NA)	No	LRYGB	No
Catheline JM - 2006 [15]	19/M	76 kg/m^2^	ECG/gastroscopy/X-ray chest/USG	NA	No	LSG	No
Genser L - 2015 [16]	52/F	49 kg/m^2^	ECG/X-ray chest/CT scan	52 min (45–60)	No	Trans-umbilical SILSG	No
Ersoy E - 2005 [17]	33/F	53 kg/m^2^	ECG/gastroscopy/X-ray chest/USG	NA	No	LAGB	No
Samaan M - 2008 [18]	29/M	56 kg/m^2^	ECG	NA	No	LAGB	Band erosion
Matar ZS - 2008 [19]	28/M	51 kg/m^2^	ECG/VFSE/X-ray chest/ USG	NA	No	LAGB	No
Taskin M - 2008 [20]	20/F	44.9 kg/m^2^	ECG/X-ray chest/USG	90 min	BIB	LAGB + LC	No
Pauli EM - 2008 [21]	47/F	60 kg/m^2^	X-ray chest/chest and abdominal CT scan	105 min	No	LAGB	No
Yazar FM - 2015 [22]	21/F	41.8 kg/m^2^	ECG/ gastroscopy/X-ray chest/USG	78 min (28–60)	No	LSG	No
Taha MH - 2017 [23]	33/F	42.7 kg/m^2^	ECG/VFSE/X-ray chest	50 min	No	LSG	No
Taha MH - 2017 [23]	41/F	41.7 kg/m^2^	ECG/VFSE/X-ray chest	75 min	No	LSG	No
Bawahab MA - 2020 [24]	30/F	36 kg/m^2^	ECG/ ECHO/X-ray chest barium meal test	28 min	Cesarean Section	LSG	No
Almussallam B - 2021 [25]	23/M	46.7 kg/m^2^	ECG/X-ray chest/Echo/CT-CAP	68 min	No	LSG	No
Villalvazo Y - 2018 [26]	59/F	38 kg/m^2^	ECHO/X-ray, CT abdomen and chest/EGD	108 min	Cesarean section, evacuation of an ectopic pregnancy	LSG	No
Poghosyan T - 2020 [27]	58/F	39 kg/m^2^	EGD	130 min	Total thyroidectomy	LRYGB	No
Burvill A - 2019 [28]	25/F	40 kg/m^2^	NA	35 min	No	LSG	No
Watanabe A - 2016 [29]	46/F	40.3 kg/m^2^	ECG/CT-CAP/EGD	202 min	Appendectomy, hysterectomy	LSG/DJB	No
Atwez A - 2018 [30]	43/F	50 kg/m^2^	NA	180 min	NA	LRYGB	No
Amirbeigi A - 2022 [31]	29/F	40 kg/m^2^	ECG/CT-CAP	75 min	No	LSG	No
Ali B - 2019 [32]	48/F	41 kg/m^2^	CT-CAP/EGD	75 min	No	LSG	No

Preoperative workups for SIT in most of the cases were ECG, X-ray chest, and abdominal CT scans. Mirroring of trocar locations and intraoperative French position were the most reported surgical techniques in LSG [8,10,15,22,24,26]. However, Deutsch et al. and Burvill et al. reported using supine position [11,28]. We managed to perform LSG with mirroring of two tracer sites and with French surgeon position. Using French position was our surgeon's preference. As per the literature and clinical experience in modern dedicated minimally invasive surgery suite, the body posture of the neck and trunk along with the orientation of the head did not differ significantly between the French and American positions [33]. In terms of tissue exposure both positions are considered to be safe and efficient depending on surgeon preference and training [33]. Interestingly, a randomized clinical trial comparing French and American positions in LSG showed the American position to have a lower physical and mental impact on the surgeon [34].

Also, it was reported that prolonged operative time and the need of additional trocar are indicators of a challenging laparoscopic procedure [22]. In our case, the operative time was similar to our regular cases and we did not require additional trocar. All reported cases in the literature were completed laparoscopically. Leakage after LSG was reported once [11]. Our patient was followed up for three months so far, and he succeeded to lose weight and did not develop any post-operative complications as reported in a handful number of cases in the literature [10,13-15,24,28].

## Conclusions

In conclusion, anatomy delineation prior to any surgical procedure is important. With good preoperative planning, performing LSG for SIT patients is safe and not technically challenging.
